# Comparative genomics analysis of mononuclear phagocyte subsets confirms homology between lymphoid tissue-resident and dermal XCR1^+^ DCs in mouse and human and distinguishes them from Langerhans cells

**DOI:** 10.1016/j.jim.2016.02.023

**Published:** 2016-05

**Authors:** Sabrina Carpentier, Thien-Phong Vu Manh, Rabie Chelbi, Sandrine Henri, Bernard Malissen, Muzlifah Haniffa, Florent Ginhoux, Marc Dalod

**Affiliations:** a Mi-mAbs (C/O Centre d'Immunologie de Marseille-Luminy), F-13009 Marseille, France; b Centre d'Immunologie de Marseille-Luminy, Aix Marseille Université UM2, Inserm, U1104, CNRS UMR7280, F-13288 Marseille Cedex 09, France; c Human Dendritic Cell Laboratory, Institute of Cellular Medicine, Newcastle University, Newcastle upon Tyne, UK; d Singapore Immunology Network (SIgN), Agency for Science, Technology and Research (A*STAR), Singapore

**Keywords:** CLN_migDCs, cutaneous lymph node migratory dendritic cells, DC, dendritic cells, DDCs, dermal dendritic cells, DMPs, dermal mononuclear phagocytes, FDR, false discovery rate, GSEA, gene set enrichment analysis, LCs, Langerhans cells, LT-DCs, lymphoid tissue-resident dendritic cells, Mac, macrophages, Mo, monocytes, MoDCs, monocyte-derived dendritic cells, MPs, mononuclear phagocytes, NES, normalized enrichment score, PCA, principal component analysis, Dendritic cells, Langerhans cells, XCR1, Skin, Comparative genomics, Bioinformatics

## Abstract

Dendritic cells (DC) are mononuclear phagocytes which exhibit a branching (dendritic) morphology and excel at naïve T cell activation. DC encompass several subsets initially identified by their expression of cell surface molecules and later shown to possess distinct functions. DC subset differentiation is orchestrated by transcription factors, growth factors and cytokines. Identifying DC subsets is challenging as very few cell surface molecules are uniquely expressed on any one of these cell populations. There is no standard consensus to identify mononuclear phagocyte subsets; varying antigens are employed depending on the tissue and animal species studied and between laboratories. This has led to confusion in how to accurately define and classify DCs across tissues and between species. Here we report a comparative genomics strategy that enables universal definition of DC and other mononuclear phagocyte subsets across species. We performed a meta-analysis of several public datasets of human and mouse mononuclear phagocyte subsets isolated from blood, spleen, skin or cutaneous lymph nodes, including by using a novel and user friendly software, BubbleGUM, which generates and integrates gene signatures for high throughput gene set enrichment analysis. This analysis demonstrates the equivalence between human and mouse skin XCR1^+^ DCs, and between mouse and human Langerhans cells.

## Highlights

• Rigorous phenotypic identification of human CD141^high^XCR1^+^ dermal DCs is challenging.
• Comparative genomics enables rigorous identification of cell populations.
• Human dermal CD141^high^XCR1^+^ DCs are homologous to human blood and mouse XCR1^+^ DCs.
• Human Langerhans cells are homologous to mouse Langerhans cells.
• Langerhans cells selectively express DC rather than macrophage gene signatures.

## 1 Introduction

Mononuclear phagocytes (MPs) comprise dendritic cells (DCs), monocytes (Mo) and macrophages (Mac). They are critical regulators of immunity, tolerance and tissue homeostasis (Guilliams et al., 2014). They populate a wide range of tissues and function as immune sentinels particularly in barrier sites such as skin, gut and lung. A thorough understanding of their development and functions is crucial to enable their manipulation for innovating immunotherapeutic and vaccination strategies.

Studies in both mouse and human have provided significant insights into MP biology; fundamentally in relation to the distinct developmental pathways and functional specializations of DC, monocyte and macrophage subsets (Dutertre et al., 2014b, Reynolds and Haniffa, 2015, Vu Manh et al., 2015a). However, the lack of a standardized definition of MP populations applicable across both tissues and species remains a pervasive challenge that hampers progress in the field (Guilliams et al., 2014). The historical definitions of MP populations based on morphology have been supplemented by surface antigen expression analysis, which over the years has undergone numerous refinements. Researchers have utilized a variety of antigen combinations and flow cytometry gating strategies to analyze MPs in different tissue and species. However, the complexity and diversity of markers used to identify subpopulations of DCs, monocytes or macrophages have prevented easy extrapolation and interpretation of published data.

The combined use of transcriptomics analysis alongside comparative biology has enabled homologous populations to be identified across tissues and species (Contreras et al., 2010, Cros et al., 2010, Crozat et al., 2010b, Crozat et al., 2011, Dutertre et al., 2014a, Gautier et al., 2012, Haniffa et al., 2012, Ingersoll et al., 2010, Marquet et al., 2014, McGovern et al., 2014, Miller et al., 2012, Reynolds and Haniffa, 2015, Robbins et al., 2008, Schlitzer et al., 2013, Segura et al., 2013, Tamoutounour et al., 2013, Vu Manh et al., 2014, Vu Manh et al., 2015a, Vu Manh et al., 2015b). However, despite the success of this strategy, non-standardized protocols to define cell populations a priori for transcriptome analysis raise questions on the comparability of findings presented between groups (Guilliams et al., 2014, Vu Manh et al., 2015a). A case in point relates to the antigens and flow cytometry gating strategies used to define dermal cross-presenting CD141^high^XCR1^+^ DCs in order to investigate their relationship to blood CD141^high^XCR1^+^ DCs and to epidermal Langerhans cells (LCs) in human. Three research groups described CD141^+/high^ DCs in the human dermis using variable flow cytometry gating strategies which resulted in three distinct populations with different transcriptome profiles and functions (Artyomov et al., 2015, Chu et al., 2012, Haniffa et al., 2012). These contrasting findings have created confusion in the field on the identification of human dermal CD141^+/high^ DCs, their functions and their homology to mouse MP subsets (Artyomov et al., 2015, Chu et al., 2012, Haniffa et al., 2012).

In this report, we demonstrate the utility of the novel BubbleGUM software (Spinelli et al., 2015), with high throughput and automated Gene Set Enrichment Analysis (GSEA) capabilities, to integrate microarray transcriptome datasets by generating gene signatures for distinct MP populations from different tissues and species for cross-comparison and identification of homologies. Using this software, as well as complementary methods including principal component analysis (PCA) and hierarchical clustering, we reconciled the inconsistencies between previous analyses of human dermal CD141^+/high^ DCs. We demonstrate that bona fide human dermal CD141^high^XCR1^+^ DCs are transcriptionally similar to human blood CD141^high^XCR1^+^ DCs and are homologous to murine dermal CD103^+^ XCR1^+^ DCs. We also confirm that human LCs are homologous to murine LCs.

## 2 Materials and methods

### 2.1 Microarray expression data

**Fig. 1:**
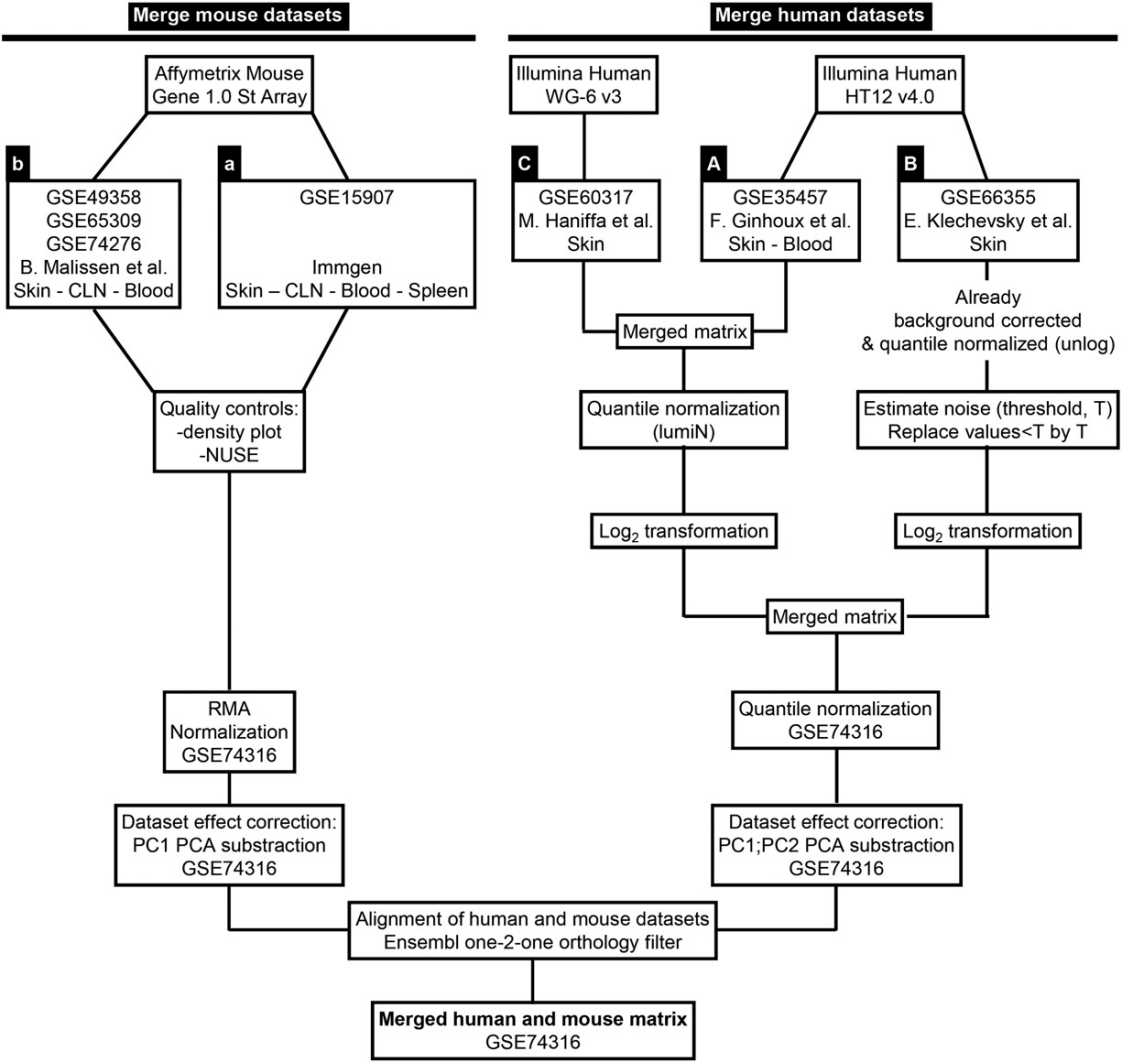
Overall scheme for the generation and analysis of datasets. Outline of pre-processing pipeline for the meta-analysis of the different mouse and human datasets analyzed.

All the microarray data used in the study were obtained from GEO database. Two datasets were used for mouse cell types and three datasets were used for human cell types (Fig. 1), to match as well as possible between tissues and species the diversity of the cell types studied. Within each species, we ensured having at least one cell type in common between the different datasets used, to enable cross-normalization and dataset effect correction. Moreover, we defined cell type-specific gene signatures in a consistent way, by comparing the cell type of interest to a set of reference cell populations that was equivalent across tissues and species. The mouse dataset “a” was generated by the Immgen consortium (GEO series identification number GSE15907). The mouse dataset “b” was generated by the Malissen laboratory (GSE49358, GSE65309 and GSE74276). Human datasets “A” and “C” were generated by the Ginhoux laboratory from blood and skin MPs (GSE35457 and GSE60317, respectively). Dataset “B” was generated by the Klechevsky laboratory from skin MPs (GSE66355). All of these data have been analyzed in previous publications, GSE15907 (Elpek et al., 2011, Gautier et al., 2012, Heng et al., 2008, Miller et al., 2012), GSE49358 (Tamoutounour et al., 2013), GSE65309 (Terhorst et al., 2015), GSE35457 (Haniffa et al., 2012), GSE60317 (McGovern et al., 2014), GSE66355 (Artyomov et al., 2015), except for a subgroup of mouse dataset b (GSE74276). The list of microarray samples used, their GEO identification numbers and associated metadata are provided in supplementary file 1. The expression matrices for the different analyses performed in this study are available in GEO under the accession number GSE74316.

### 2.2 Generation of the microarray compendia and their analyses by PCA or hierarchical clustering

The methodological procedure for the preprocessing of the different microarray datasets used in the study for meta-analysis is described in Fig. 1.

**Fig. S1:**
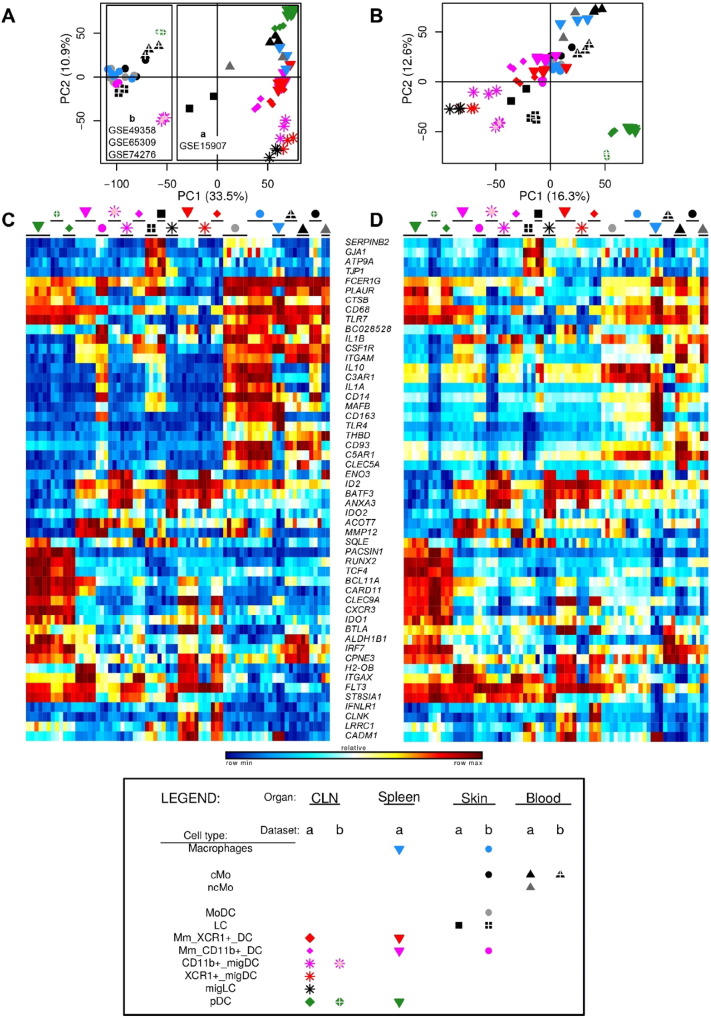
Expression patterns of control genes showing that subtraction of PC1 from the merged mouse datasets did not confound major transcriptomic characteristics of MP subsets. PCA plots (A, B) and heatmaps showing expression pattern of 52 control genes previously known to be differentially expressed between mouse MP subsets (C, D) before (A, C) and after (B, D) dataset effect removal in the mouse compendium.

The mouse Gene 1.0 ST CEL files were processed through Bioconductor in the R statistical environment (version 3.0.2). Quality control of the array hybridization (NUSE plot) and normalization of the raw Affymetrix expression data with Robust Multi-chip Analysis (Irizarry et al., 2003) were performed using the oligo package. PCA was performed using ade4 package to remove the dataset effect visible on the first principal component (Fig. S1A–B). The two mouse datasets included in common epidermal LCs, blood classical monocytes (cMo), cutaneous lymph node (CLN) CD11b^+^ migratory DCs (migDCs) and CLN plasmacytoid DCs (pDCs). For each of these control cell populations, the samples from the two datasets regrouped well together in the PCA or hierarchical clustering analyses after merging and correction of the dataset effect. Similar expression patterns were observed within each mouse dataset before and after data merging, quantile cross-normalization and dataset effect removal, when examining 52 control genes encoding key lineage-specific transcription factors, innate immune recognition receptors or signaling molecules (Fig. S1C–D). This validated the adequacy of data preprocessing to prevent artefactual changes in the relative gene expression between cell populations or datasets.

**Fig. S2:**
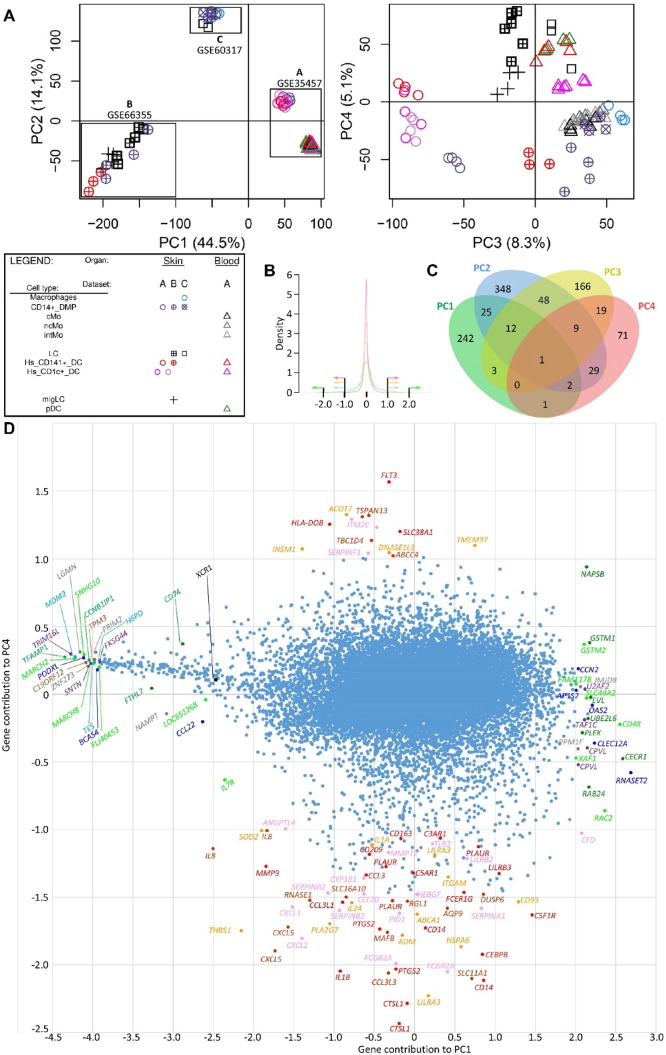
Identification of the genes contributing the most to dataset effect (PC1) or to DC versus monocyte/macrophage identity (PC4) for the human compendium. (A) PCA plots before correction for dataset effect. (B) Distribution of the weights of individual genes for the complete human dataset. Distributions of weights are shown as individual colored curve for each of the 4 PC axes, in green for PC1, blue for PC2, yellow for PC3 and red for PC4, and were used to set up threshold for selection of the genes contributing the most to each PC axis as shown by the colored arrows on the graph. (C) Venn diagram showing the intersections between the lists of the genes contributing the most to each of the first four PC axis. Only 4 of the 132 genes contributing the most to PC4 also strongly contribute to PC1. The vast majority of the genes contributing the most to PC1 and PC2 do not strongly contribute to PC3 or PC4. (D) Dot plot showing the individual contribution of each gene to PC1 (X-axis) versus PC4 (Y-axis). Most of the genes strongly contributing to PC1 do not significantly contribute to PC4 and are not known to be involved in the biology of DC or monocyte/macrophage subsets. Conversely, most of the genes contributing strongly to PC4 do not strongly contribute to PC1, and many of these genes are known to be specifically expressed by, or to control the development or functions of, DC or monocyte/macrophage subsets.

**Fig. S3:**
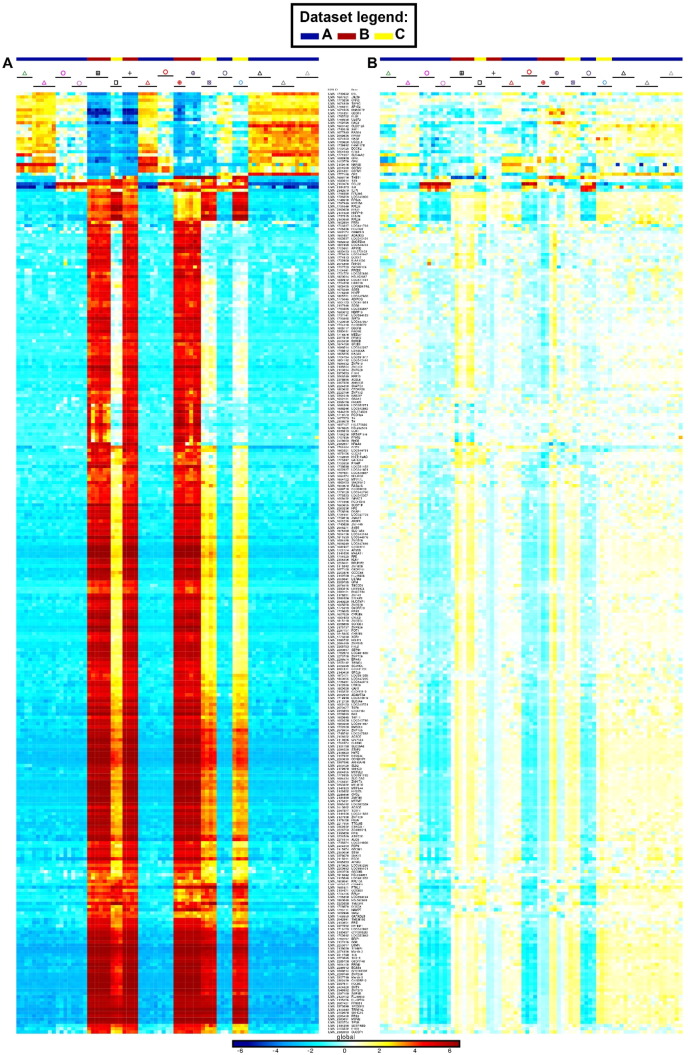

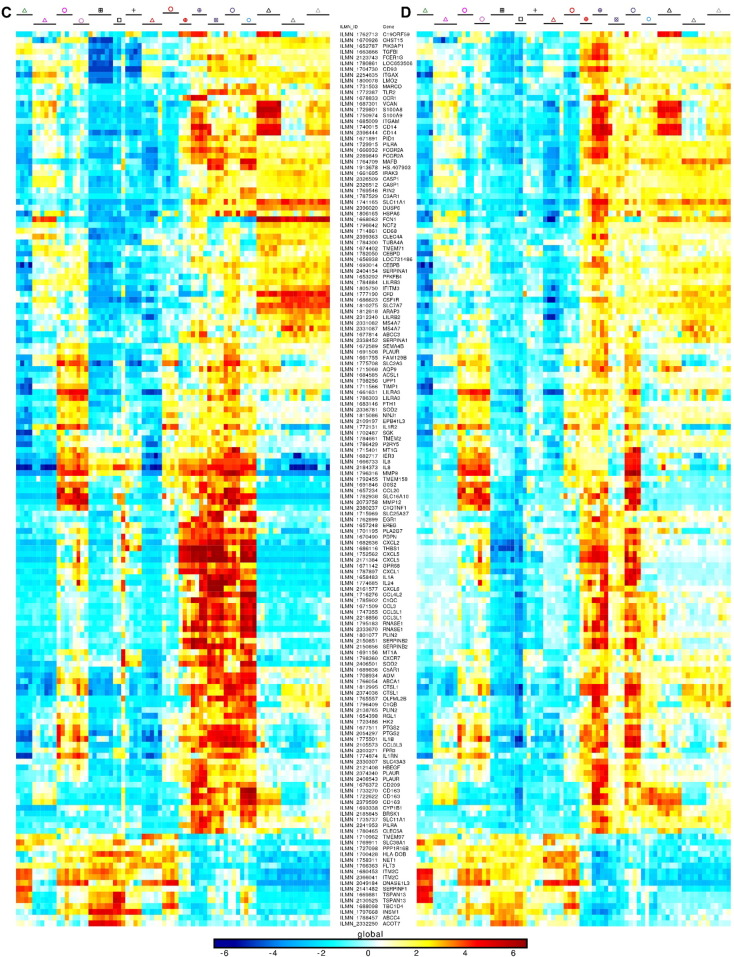
Removal of gene contributions to PC1 (dataset effect) and PC2 (dataset and tissue effect) does not confound relationships between human DC versus monocyte/macrophage subsets. (A–B). Heatmap showing the expression pattern of the genes contributing the most to PC1 before (A) or after (B) dataset correction. (C–D) Heatmap showing the expression pattern of the genes contributing the most to PC4 before (C) or after (D) dataset correction. The lists of genes were defined as shown in Fig. S2B–C. Before dataset correction, the PC1 genes are mostly differentially expressed between datasets but much less across DC and monocyte/macrophage subsets within each dataset. Consistently, correction of dataset effect leads to only very differential expression of these genes across the entire dataset. Before dataset correction, the PC4 genes are strongly differentially expressed within each dataset between DC and monocyte/macrophage subsets. Consistently, correction of dataset effect preserves the differential expression of these genes between DC and monocyte/macrophage subsets within and between datasets.

**Fig. S4:**
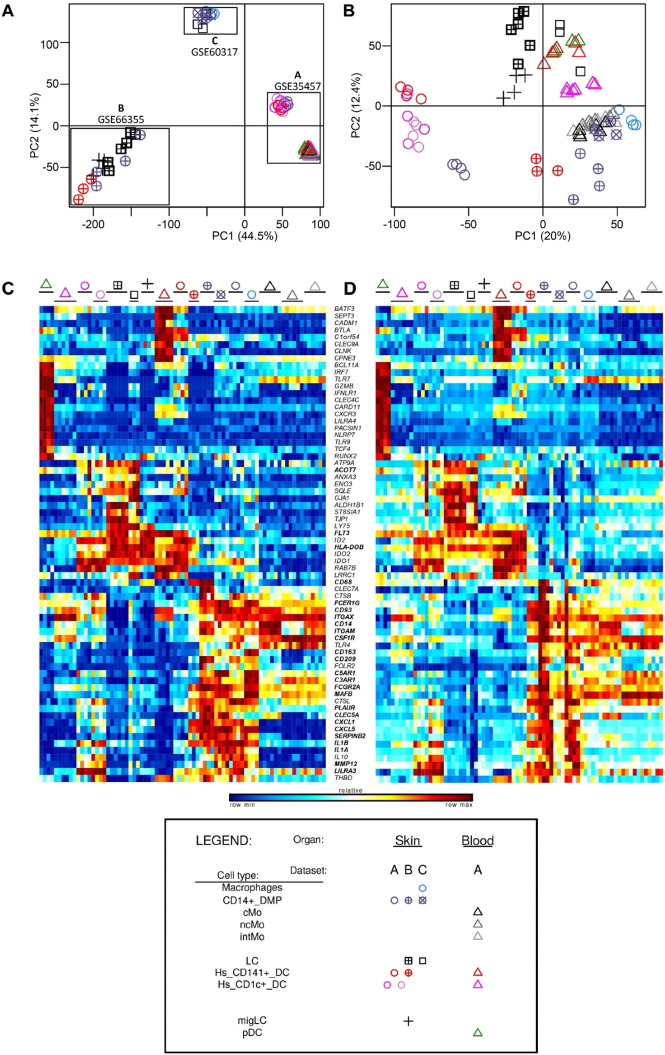
Expression patterns of control genes showing that subtraction of PC1 and PC2 from the merged human datasets did not confound major transcriptomic characteristics of MP subsets. PCA plots (A, B) and heatmaps showing expression pattern of 68 control genes previously known to be differentially expressed between mouse MP subsets (C, D) before (A, C) and after (B, D) dataset effect removal in the human compendia.

The Illumina Human WG-6 v3 and Illumina Human HT12 v4.0 raw data files were processed through Bioconductor in the R statistical environment (version 3.0.2). Gene expression signals from GSE60317 (Haniffa et al., 2012) and GSE35457 (McGovern et al., 2014) were merged using common probes. Quantile Normalization (Bolstad et al., 2004) was applied on the merged expression arrays using the lumi package, prior to log_2_-transformation of expression values. Gene expression signals from GSE66355 were already background corrected and quantile normalized (Artyomov et al., 2015). Noise threshold was estimated at five based on the density of all gene expression signals. All values less than five were replaced by this noise threshold. Expression values were log_2_-transformed to enable comparison with the other two human microarray datasets. The three datasets were merged using common probes and quantile normalized. PCA was performed to remove the dataset effect visible on the first two principal components (Figs. S2, S3 and S4). CD14^+^ dermal MPs (CD14^+^_DMPs) was a common population encompassed in all three datasets. In addition, both datasets B and C contained epidermal LCs. LC samples regrouped together in PCA or hierarchical clustering after the datasets were merged and corrected. This was also the case for CD14^+^_DMPs from datasets B and C but the CD14^+^_DMPs from dataset A was more distant. However, when examining individual gene contributions to PCA axes on the merged datasets before correction (Fig. S2B–D), the vast majority of transcripts accounting for the differences between datasets according to PC1 and PC2 did not contribute significantly to differences between MP subsets according to PC3 and PC4. Many genes known as selectively expressed by, or affecting the biology of, MP subsets were strong contributors to PC4 but had only a weak contribution to PC1 (Fig. S2D). In contrast, the vast majority of the transcripts strongly contributing to PC1 were not known to affect the identity or biology of MP subsets, and were not differentially expressed between subsets of DCs or monocytes/macrophages within each dataset (Fig. S3). Careful scrutiny of the expression patterns of 68 genes encoding key lineage-specific transcription factors, innate immune recognition receptors or signaling molecules within each human dataset before and after data merging, quantile cross-normalization, and dataset effect correction revealed no obvious biases or artifacts from data pre-processing (Fig. S4). In particular, CD14^+^_DMPs in all three human datasets expressed high levels of monocyte/macrophage genes and low levels of genes specific to other cell lineages, before and after data processing. Hence, data preprocessing was adequate and did not cause artefactual changes in the relative gene expression profiling between cell populations or datasets which could confound the subsequent analyses and their interpretation.

Human and Mouse datasets were then merged into a single matrix of orthologous genes identified using the Ensembl BioMart software with selection of “one-to-one” orthology relationships only (13,371 unique genes). Agglomerative hierarchical clustering (using Hmisc and cluster packages) and PCA (using ade4 R package) were performed on the merged human and mouse matrix. We used distinct distance metric/linkage parameters to generate several agglomerative hierarchical clustering trees, because this approach can reveal distinct similarity patterns in the data.

### 2.3 Generation of gene signatures and high throughput GSEA using BubbleGUM

The BubbleGUM software (Spinelli et al., 2015) was used to generate MP subset-specific transcriptomic signatures from specific datasets and to assess their enrichment across cell types from other datasets. BubbleGUM is an open-source software composed of two modules; i) GeneSign, which generates statistically significant gene signatures and ii) BubbleMap, which automatically assesses the enrichment of input gene signatures between all possible pairs of conditions from independent datasets, based on gene set enrichment analysis (GSEA) methodology (Subramanian et al., 2005, Subramanian et al., 2007), and which generates an integrated graphical display.

Using GeneSign, gene signatures of MP subsets were generated for each species (human and mouse), i.e. the lists of genes that are more highly expressed in the MP subsets of interest (test classes) as compared to other MP subsets (reference classes), using the “minimal pairwise mean” calculation method with a minimal fold change of 1.5 in linear scale and a maximal false discovery rate (FDR) of 0.01. The test and reference classes of MP subsets chosen to define each of the cell type signatures used in the manuscript, as well as the gene content of these signatures, are provided in supplementary file 1.

To run BubbleMap, we used in-house signatures of mouse or human MP subsets devised in GeneSign as well as a list of over 400 independent GeneSets from MSigDB (Liberzon, 2014). BubbleMap was used with 1000 geneset-based permutations, and with “difference of classes” as a metric for ranking the genes since the data were expressed in Log_2_ scale. The results are displayed as a BubbleMap, where each bubble is a GSEA result and summarizes the information from the corresponding enrichment plot. The color of the Bubble corresponds to the subset from the pairwise comparison in which the signature is enriched. The bubble area is proportional to the GSEA normalized enrichment score (NES). The intensity of the color corresponds to the statistical significance of the enrichment, derived by computing the multiple testing-adjusted permutation-based p-value using the Benjamini–Yekutieli correction.

### 2.4 Heatmaps

Heatmaps were performed using Gene-E (http://www.broadinstitute.org/cancer/software/GENE-E/), using the final gene expression values for the human and mouse datasets after merging the various datasets into a single matrix, with removal of the first (or first two) principal component(s) of PCA which generated negative expression values. The expression values were not centered and reduced, in order to avoid misrepresenting the relative importance of individual genes by changing their variance. This allowed retention of genes with high variance in only one species but not the other. For the same rationale, global scale expression values were used for graphical output, rather than relative scales adjusted for each gene to its minimal and maximal expression values across the entire dataset.

## 3 Results

### 3.1 Human and mouse LCs transcriptionally resemble DCs

**Fig. 2:**
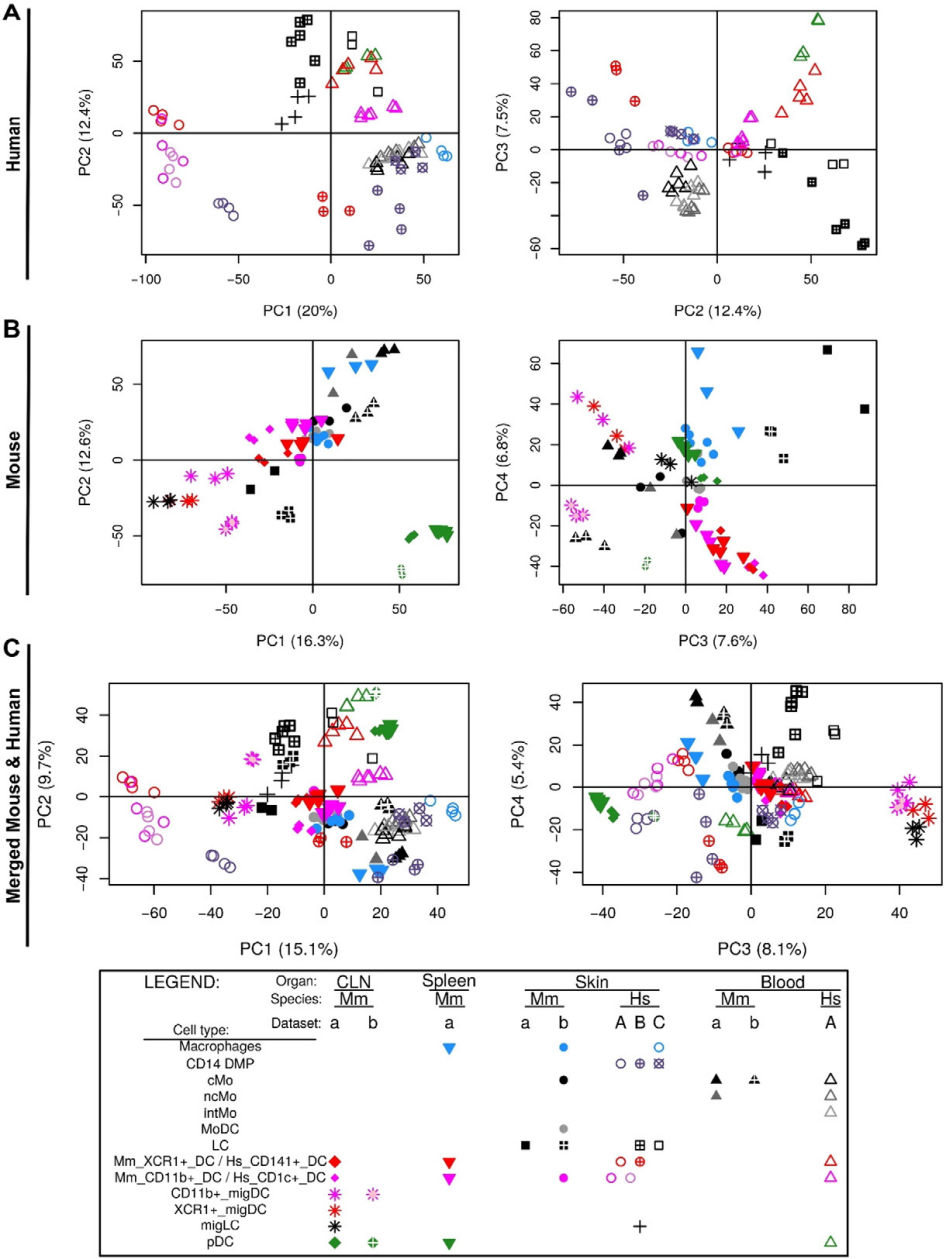
Relationships between MP subsets by PCA. Principal component analysis of human MP subsets (A), mouse MP subsets (B) and merged human and mouse MP subsets (C). Numbers in parenthesis indicate the percentage variability of the dataset along each PC axis.

**Fig. 3:**
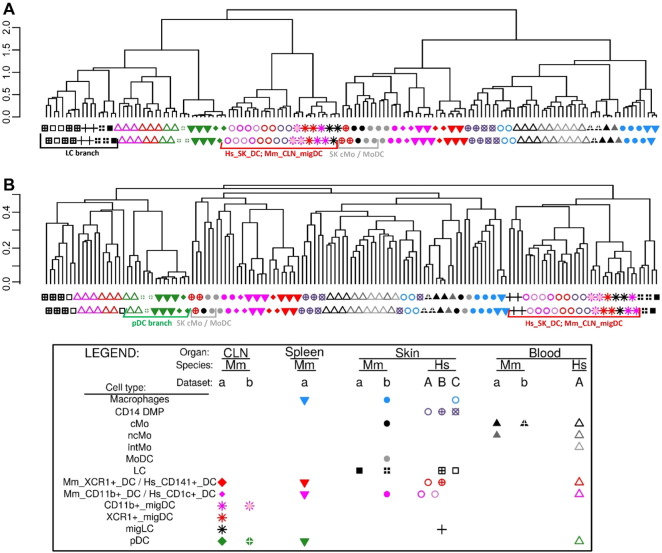
Relationships between MP subsets by hierarchical clustering. (A) Pearson correlation distance and Ward's method linkage. (B) Pearson correlation distance and average linkage.

Within the human compendium (Fig. 2A), LCs regrouped with blood DCs in the upper half of PC1 vs PC2, while blood Mo, skin Mac (SK_Mac) and skin CD14^+^_DMPs (SK_CD14^+^_DMPs) regrouped together in the lower right quadrant. Within the mouse compendium (Fig. 2B), LCs also regrouped with DCs, in the lower left quadrant of PC1 vs PC2, while blood and skin monocytes regrouped with skin macrophages and monocyte-derived DCs (MoDCs), in the upper right quadrant. For hierarchical clustering, various parameters were used for distance calculation to generate several trees revealing distinct similarity patterns in the data. In most hierarchical clustering analyses, mouse and human LCs regrouped with subsets of cDCs and were excluded of the branch of the tree encompassing all monocytes or monocyte-derived cells (Fig. 3A). In the hierarchical clustering analyses where mouse and human LCs did not regroup together, they were still associated with subsets of cDCs and not with monocytes or monocyte-derived cells (Fig. 3B).

**Fig. 4:**
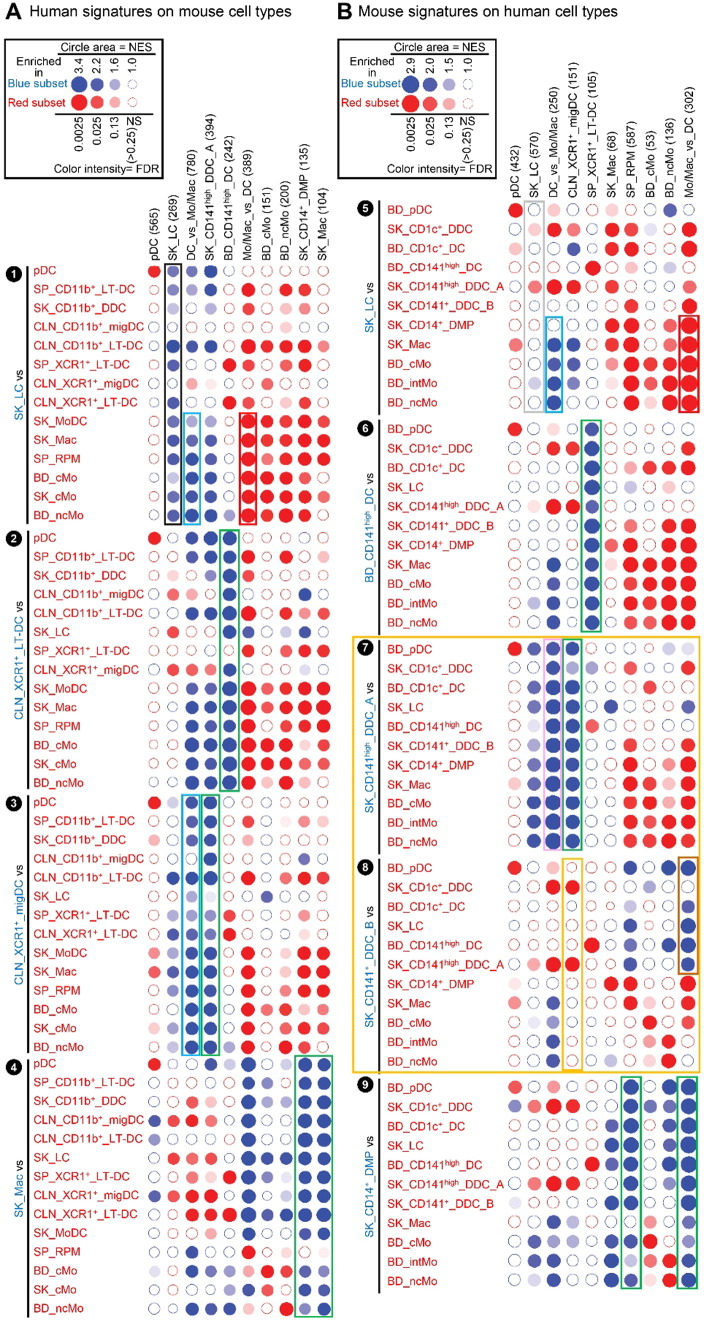
Analysis of the homologies between human and mouse MP subsets by high throughput GSEA using BubbleGUM. Gene signatures specific to each subset of MPs, or their subgroups, were generated using GeneSign separately for the mouse and human compendia. These signatures obtained in one species were then assessed for enrichment in all possible pairwise comparisons between MP subsets of the other species using the BubbleMap module of BubbleGUM. Data are represented as Bubbles, bigger and darker for stronger and more significant enrichment, in a color matching that of the condition in which the GeneSet was enriched (blue for the population indicated in blue characters on the annotation on the left of each figure, red for the populations to which the comparison is performed). The strength of the enrichment is quantified by the NES which represents the number and differential expression intensity of the genes enriched. The significance of the enrichment is measured by the false discovery rate (FDR) value (q) representing the likelihood that the enrichment of the GeneSet was a false-positive finding (e.g., if q = 0.25, a similar enrichment is found in 25% of the random GeneSets used as controls). This q-value was further corrected for multiple testing, leading to a higher stringency of the significance threshold used. The absolute NES values generally vary between 1 (no enrichment) and 5 (extremely high enrichment). The enrichment is considered significant for absolute NES values > 1 with an associated q value < 0.25. (A) Human MP signatures assessed for enrichment across mouse MP subsets. (B) Mouse MP signatures assessed for enrichment across human MP subsets.

**Fig. 5:**
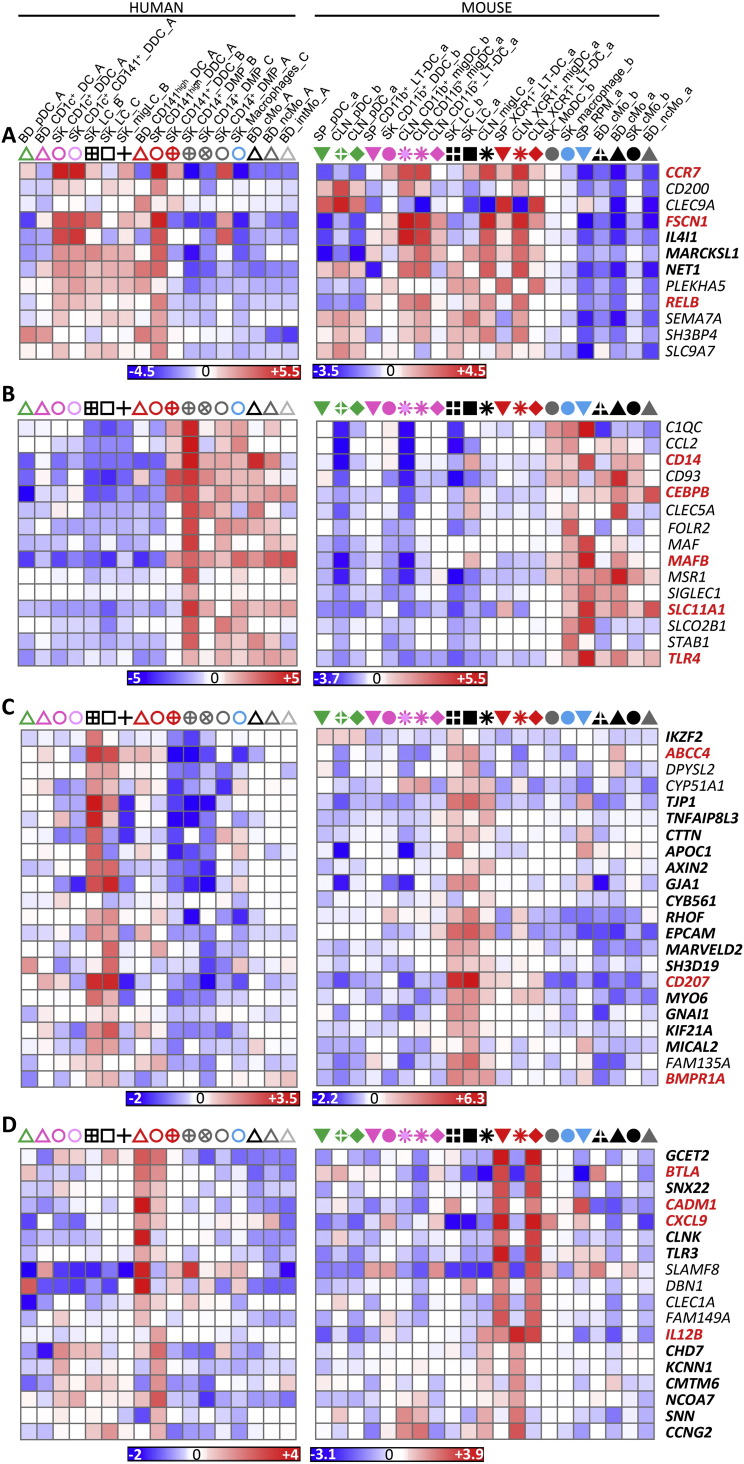
Heatmaps of selected genes contributing to GSEA profiles in Fig. 4. Expression data collapsed to the median expression across replicates are shown for the human (left) and mouse (right) compendia. Each cell type is depicted by the same symbol used in the PCA in Fig. 2, with the name of cell types spelled out above the figure. (A) Genes from the mouse and human cDC_vs_Mo/Mac GeneSet. (B) Genes from the mouse and human Mo/Mac_vs_DC GeneSet. (C) Genes from the mouse and human SK_LC GeneSets. (D) Genes from the human BD_CD141^high^_DC and/or SK_CD141^high^_DDC_A GeneSets and from the mouse SP_XCR1^+^_LT-DC and/or CLN_XCR1^+^_migDC GeneSets. Genes regulating the development or functions of the MP subset(s) in which they are selectively expressed are shown in bold red font. Genes for which a selective expression pattern was previously and independently reported across several subsets of mouse or human MPs, with results consistent with those shown here, are in bold black font.

High throughput GSEA performed using our BubbleGUM software (Spinelli et al., 2015) showed that, when compared to each of the populations of monocytes or monocyte-derived cells, mouse and human LCs were enriched for the genes more highly expressed in DCs compared to monocytes/macrophages (DC_vs_Mo/Mac gene signatures) (Fig. 4, ❶, ❺, blue boxes). Conversely, the reciprocal Mo/Mac_vs_DC gene signatures were enriched in each of the populations of mouse or human monocytes or monocyte-derived cells analyzed when they were compared to LCs (Fig. 4, ❶, ❺, red boxes). Expression profiles of genes contributing to these GSEA patterns are depicted as heatmaps of genes with higher expression in cDCs and LCs (Fig. 5A) and of genes with higher expression in monocytes and monocyte-derived cells (Fig. 5B).

### 3.2 Human and mouse LCs are homologous and express common gene signatures

Human and mouse LCs on the one hand, and mouse and human pDCs on the other hand, regrouped together within the merged human/mouse compendia by PCA, on the PC1 versus PC2 representation (15.1% versus 9.7% variability, respectively) (Fig. 2C). The use of different linkage methodologies for hierarchical clustering revealed distinct similarity patterns in the data, since human and mouse LCs but not mouse and human pDCs clustered together in most instances (Fig. 3A), whereas the reverse was observed in other instances (Fig. 3B), consistent with the observation that human and mouse pDCs were separated on the PC3 axis (8.1% variability) and mouse and human LCs on the PC4 axis (5.4%) of the PCA (Fig. 2C). Mouse LCs were significantly enriched for the human LC gene signature by GSEA (Fig. 4, ❶, black box). This enrichment does not reach significance only for the comparison with CLN migratory cDC subsets. Genes selectively expressed in both mouse and human LCs are shown as a heatmap, including genes that were used to define LCs in human and/or mouse (*CD207*, *EPCAM*), selectively expressed in human LCs compared to other skin MP subsets (Polak et al., 2014), or regulating LC functions (*ABCC4* and *BMPR1A*) (van de Ven et al., 2008, Yasmin et al., 2013) (Fig. 5C).

These analyses show that, contrary to what was recently reported (Artyomov et al., 2015), human LCs do not display a greater similarity to the mouse XCR1^+^ DCs than to mouse LCs. Rather, human and mouse LCs are homologous and express a common gene signature.

### 3.3 The identification of bona fide human CD141^high^XCR1^+^ dermal DCs is challenging

A dermal DC subset homologous to human blood CD141^high^XCR1^+^ DCs has been identified in the human dermis and shown to be very efficient at antigen cross-presentation similar to their murine counterparts (Haniffa et al., 2012). Other teams have studied human dermal CD141^+^ DCs but reported different functional properties and homology relationships (Artyomov et al., 2015, Chu et al., 2012). A recent report claimed that human LCs and not SK_CD141^+^_DDCs were equivalent to human blood CD141^high^XCR1^+^ DCs and to mouse XCR1^+^ DDCs (Artyomov et al., 2015). As CD141 is promiscuously expressed on various MP subsets (Crozat et al., 2010a, Haniffa et al., 2012, McGovern et al., 2014, Vu Manh et al., 2015a), we wondered whether the discrepancies existing in the literature on the functions of dermal CD141^high/+^ DCs could be due to variations in the flow cytometry gating strategies used to identify these cells resulting in different CD141^+/high^ populations being studied. The CD141^+^ DCs isolated by Chu et al. expressed the monocyte marker CD14 and corresponded to CD14^+^ dermal monocyte-derived macrophages (McGovern et al., 2014). The identity of the CD141^+^ dermal cells studied by Artyomov et al. was less obvious, although they were CD14^−^ and CD1a^low^ and stained with an anti-XCR1 antibody. Interestingly, the three isolation strategies resulted in dermal CD141^+/high^ DCs with contrasting transcriptome profiles.

In order to further assess the identity and lineage relationships of the two reported dermal CD141^high/+^ DCs, we reanalyzed the human DC, monocyte and macrophage microarray compendia associated with these studies (Artyomov et al., 2015, Haniffa et al., 2012, McGovern et al., 2014). Of note, all three datasets included CD14^+^_DMPs, and LCs were shared between the Artyomov and McGovern datasets, allowing to control dataset compatibility as discussed in Section 2. The SK_CD14^+^_DMPs shared similar transcriptomic characteristics even though differences linked to datasets of origin existed. In PCA and hierarchical clustering, human epidermal LCs (SK_LCs) regrouped together irrespective of their dataset of origin (Fig. 2, Fig. 3). In contrast, strikingly, the two sets of skin CD141^+/high^_DDCs from Haniffa et al., and Artyomov et al., clustered in very different areas by PCA (Fig. 2A-B) and hierarchical clustering (Fig. 3A-B). By PCA, SK_CD141^high^_DDCs_A from the pioneering publication which reported their discovery (Haniffa et al., 2012) were close to SK_CD1c^+^_DDCs and distant from SK_CD14^+^_DMPs, while the converse was observed for the SK_CD141^+^_DDCs_B described in the publication which reported lack of close homology of these cells with human BD_CD141^high^_DCs and mouse XCR1^+^ DCs (Artyomov et al., 2015). By hierarchical clustering (Fig. 3), the SK_CD141^+^_DDCs_B clustered together with mouse SK_MoDCs. These observations strongly suggested that the SK_CD141^high^_DDCs_A were related to DCs, whereas the SK_CD141^+^_DDCs_B were a subset of monocyte-derived cells.

We next performed GSEA to compare human CD141^high/+^ DCs and mouse XCR1^+^ DCs. The gene signature of the human SK_CD141^high^_DDC_A was significantly enriched in the mouse CLN_XCR1^+^_migDCs (Fig. 4A, ❸, green box). Reciprocally, the gene signature of the mouse CLN_XCR1^+^_migDCs was significantly enriched in human SK_CD141^high^_DDCs_A (Fig. 4B, ❼, green box). In contrast, the gene signature of human SK_CD141^+^_DDC_B was not significantly enriched in any of the mouse DC subsets examined (not shown). In addition, the human SK_CD141^+^_DDCs_B were not significantly enriched for the gene signature of the mouse CLN_XCR1^+^_migDCs when compared to any of the other 11 human cell subsets studied (Fig. 4B, ❽, orange box). In contrast to human SK_CD141^high^_DDCs_A which were enriched for the mouse DC_vs_Mo/Mac signatures (Fig. 4B, ❼, pink box), SK_CD141^+^_DDCs_B were enriched for the mouse Mo/Mac_vs_DC signatures when compared to all previously characterized human skin or blood DC subsets, including SK_CD141^high^_DDCs_A, BD_CD141^high^_DCs, BD_pDCs, BD_CD1c^+^_DCs and SK_LC (Fig. 4B, ❽, brown box). Comparing SK_CD141^high^_DDCs_A and SK_CD141^+^_DDCs_B individually against all other human MP subsets revealed the striking difference between these two populations (orange box, Fig. 4B). This highlights the major drawback of a priori population definition using different flow cytometry gating strategies.

Genes contributing to the GSEA profiles from the human and mouse compendia are shown as heatmaps (Fig. 5). Genes more highly expressed in cDCs include hallmark genes of mature DCs (*CCR7*, *RELB*, *FASCIN1*, *IL4I1*, *MARCKSL1*) (Dalod et al., 2014, Miller et al., 2012, Vu Manh et al., 2013) which were also highly expressed in SK_CD141^high^_DDCs_A over Mo/Mac and SK_CD141^+^_DDCs_B (Fig. 5A). This is in contrast to SK_CD141^+^_DDCs_B, which expressed genes characteristic of Mo/Mac and/or regulating their development (*CEBPB*, *MAFB*, *CD14*, *TLR4*, *SLC11A1*) (Cain et al., 2013, Gautier et al., 2012, Kelly et al., 2000, Wyllie et al., 2002) (Fig. 5B). Genes highly expressed commonly in human SK_CD141^high^_DDCs_A and mouse CLN_XCR1^+^_migDCs or mouse XCR1^+^_LT-DCs encompassed many genes previously reported to be characteristic of this cross-presenting DC subset (*BTLA*, *CADM1*, *CXCL9*, *GCET2*, *IL12B*, *SNX22* and *TLR3*) (Alexandre et al., 2016, Balan et al., 2014, Dutertre et al., 2014a, Galibert et al., 2005, Haniffa et al., 2012, Lee et al., 2015, Meixlsperger et al., 2013, Poulin et al., 2010, Robbins et al., 2008, Shortman and Heath, 2010, Vu Manh et al., 2015a, Vu Manh et al., 2015b) (Fig. 5D). Notably, a number of these genes were strongly downregulated in mouse CLN_XCR1^+^_migDCs as previously reported, as a consequence of their maturation (Crozat et al., 2011, Vu Manh et al., 2013) or of their imprinting by the skin microenvironment. A similar downregulation of the canonical gene signature of the cross-presenting DC subset was also observed in human SK_CD141^high^_DDC_A (Fig. 5D, see for example *CADM1*, *CLNK* and *TLR3*, and data not shown). This may have contributed to make their rigorous identification more difficult.

**Fig. S5:**
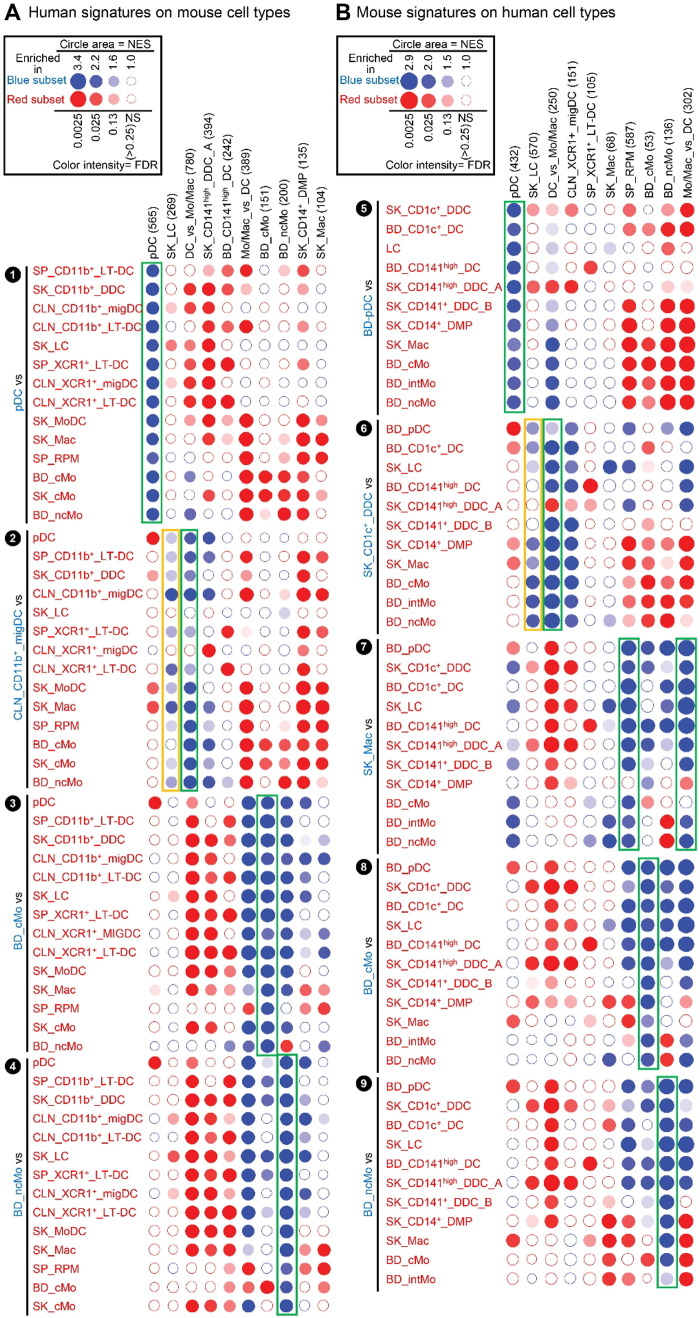
Analysis of the homologies between human and mouse MP subsets by high throughput GSEA using BubbleGUM. Additional comparisons between cell subsets to complete the analysis shown in Fig. 4. Gene signatures specific to each subset of MPs, or their subgroups, were generated using GeneSign separately for the mouse and human compendia. These signatures obtained in one species were then assessed for enrichment in all possible pairwise comparisons between MP subsets of the other species using BubbleMap. Data are represented as Bubbles, bigger and darker for stronger and more significant enrichment, in a color matching that of the condition in which the GeneSet was enriched (blue for the population indicated in blue characters on the annotation on the left of each figure, red for the populations to which the comparison is performed). (A) Human MP signatures assessed for enrichment across mouse MP subsets. (B) Mouse MP signatures assessed for enrichment across human MP subsets. Green boxes correspond to expected control enrichments, where a cell type-specific gene signature in one species is enriched in the homologous cell population in the other species when compared to any other cell population.

For each known pair of homologous mouse and human MP subset, a very strong, specific and significant enrichments of the corresponding gene signatures from one species was also observed in the other species, providing excellent positive and negative controls for the BubbleGUM analysis. These included the enrichment of the following gene signatures: human BD_CD141^high^_DC in mouse CLN_XCR1^+^_LT_DC (Fig. 4, ❷), human SK_CD14^+^_DMP and SK_Mac in mouse SK_Mac (Fig. 4, ❹), mouse Mo/Mac_vs_DC and SP_RPM in human SK_CD14^+^_DMPs (Fig. 4, ❾), human pDC in mouse pDCs (Fig. S5A, ❶) and reciprocally mouse pDC in human pDCs (Fig. S5B, ❺). Furthermore, using BubbleGUM, we confirmed previous reports of homology between human and mouse monocytes subsets (Fig. S5, ❸, ❹, ❽, ❾) (Cros et al., 2010, Ingersoll et al., 2010, Vu Manh et al., 2015b).

**Fig. S6:**
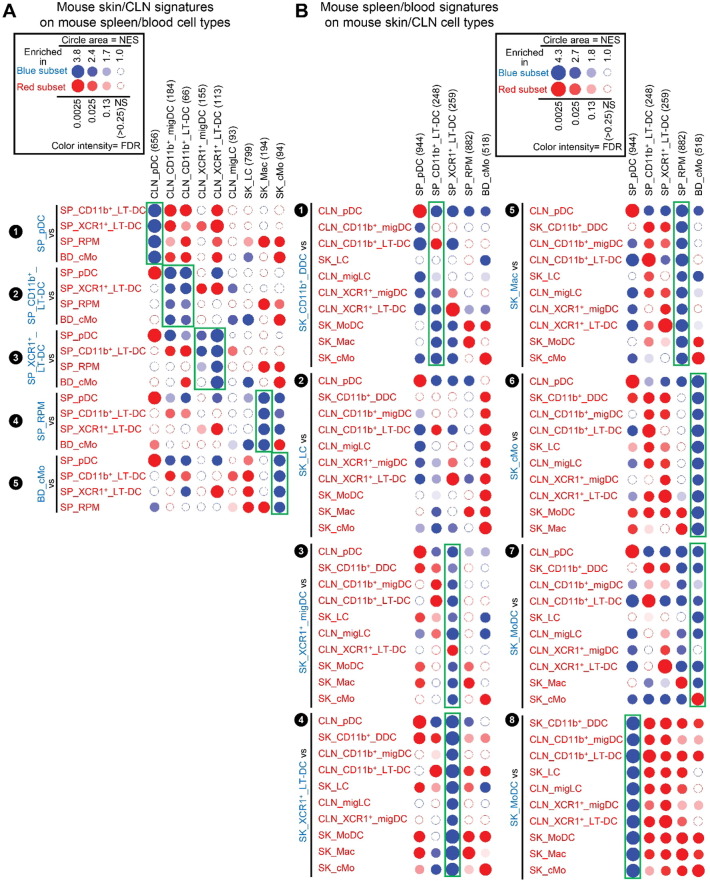
Analysis of the homologies between mouse skin/CLN versus spleen/blood MP subsets by high throughput GSEA using BubbleGUM. The analysis was performed and represented as explained in the legend of Fig. 6, but for the mouse dataset instead of the human one. Gene signatures specific to each subset of mouse MPs, or to subgroups of MPs, were generated independently from skin/CLN versus spleen/blood data using GeneSign. The signatures obtained in one type of tissue were assessed for enrichment in all possible pairwise comparisons between MP subsets from the other type of tissue using BubbleMap. Data are represented as in Fig. 4. (A) Mouse skin/CLN MP gene signatures assessed for enrichment across mouse spleen/blood MP subsets. (B) Mouse spleen/blood MP gene signatures assessed for enrichment across mouse skin/CLN MP subsets. Green boxes correspond to expected control enrichments, where a cell type-specific signature in one tissue is enriched in the equivalent cell population in the other tissue when compared to any other cell population.

**Fig. 6:**
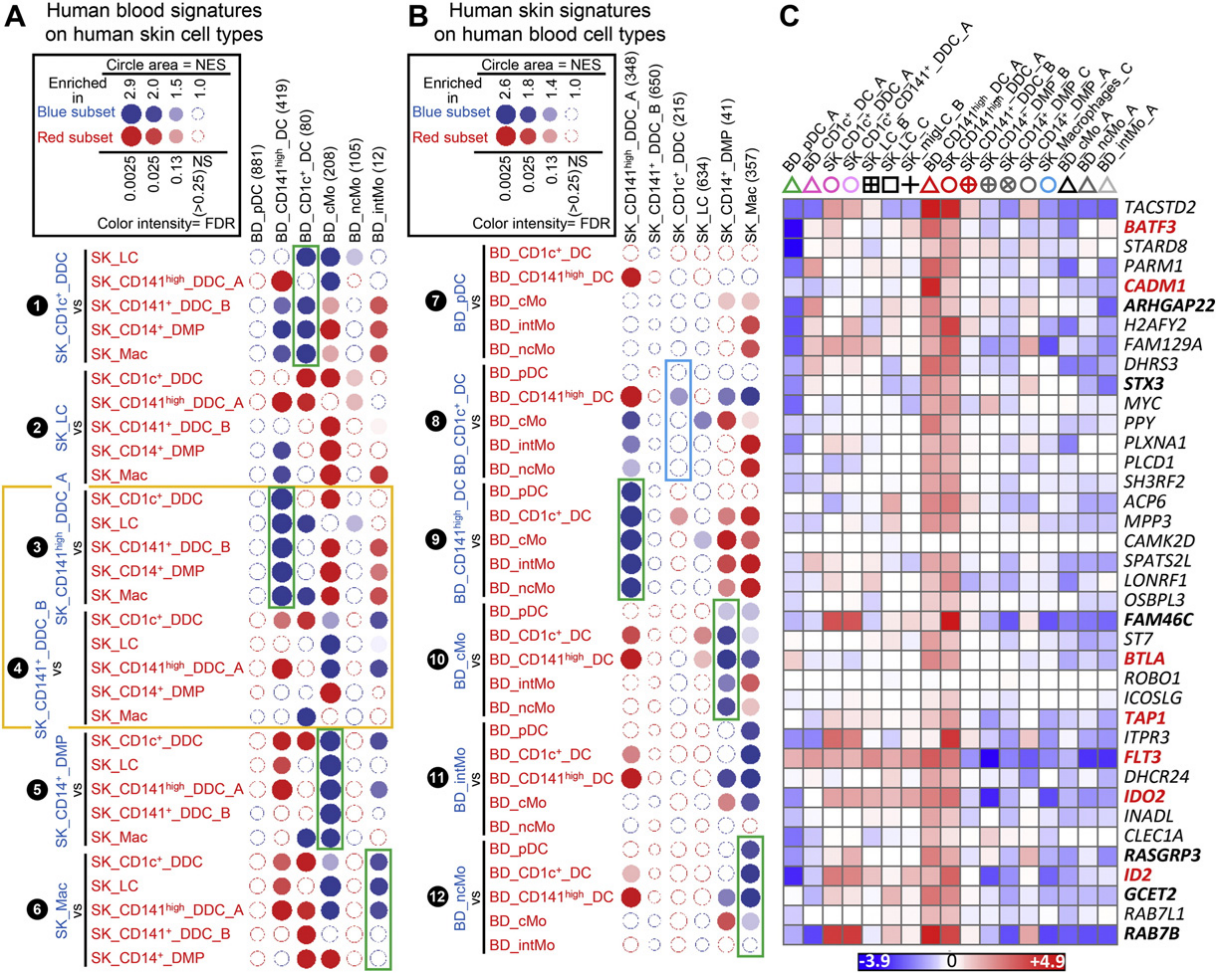
Analysis of the homologies between human blood and skin MP subsets by high throughput GSEA using BubbleGUM. Gene signatures specific to each subset of human MPs, or to subgroups of MPs, were generated independently from blood and skin data using GeneSign. The signatures obtained in one tissue were assessed for enrichment in all possible pairwise comparisons between MP subsets from the other tissue using BubbleMap. Data are represented as in Fig. 4. (A) Human blood MP gene signatures assessed for enrichment across human skin MP subsets. (B) Human skin MP gene signatures assessed for enrichment across human blood MP subsets. (C) Heatmaps illustrating the expression patterns of selected genes contributing to the GSEA profiles of (A) and (B). Expression data were collapsed to the median expression across replicates within the human compendium. Each cell type is depicted by the same symbol used in the PCA in Fig. 2, with the name of cell types spelled out above. Genes previously reported to be characteristic of this DC subset in human or mouse are in bold black font, and genes known to control their development or functions in bold red font.

In order to identify the tissue equivalents of blood CD141^high^ DCs, we generated gene signatures of human blood MP subsets and analyzed their enrichment across human skin MPsubsets (Fig. 6). This confirmed our prediction that only SK_CD141^high^_DDCs_A but not SK_CD141^+^_DDCs_B were the tissue equivalent of blood CD141^high^_DCs (Fig. 6A, ❸, green box). SK_CD141^high^_DDCs_A and SK_CD141^+^_DDCs_B were enriched with gene signatures from distinct blood MP populations (orange box, Fig. 6A) providing further support of their distinct identities. The human BD_CD141^high^_DC gene signature was significantly enriched in the human SK_CD141^high^_DDC_A when compared to each of the other human skin MP subsets examined, including SK_LCs and SK_CD141^+^_DDC_B (Fig. 6A; ❸, green box), as previously reported using the CMAP method (Haniffa et al., 2012). In contrast, none of the human blood MP signature was consistently enriched in SK_CD141^+^_DDC_B when compared to all other human skin MP subsets (Fig. 6A, ❹). Similarly, when analyzing across human blood cell subsets the expression of the gene signatures of human skin cell subsets, the SK_CD141^high^_DDC_A signature was significantly enriched in the human BD_CD141^high^_DC (Fig. 6B, ❾, green box). In contrast, the SK_CD141^+^_DDC_B signature was not found to be significantly enriched in any of the human blood cell subsets examined. Genes selectively expressed in both BD_CD141^high^_DCs and SK_CD141^high^_DDC_A include genes previously reported to be characteristic of this DC subset in human or mouse, eventually controlling their development or functions (Fig. 6C) (*BATF3*, *CADM1*, *GCET2*, *IDO2*, *RAB7B*, *ID2*, *BTLA*, *STX3*, *FAM46C*, *TAP1*, *ARHGAP22*, *RASGPR3*) (Galibert et al., 2005, Ginhoux et al., 2009, Grajales-Reyes et al., 2015, Hacker et al., 2003, Haniffa et al., 2012, Hildner et al., 2008, Jackson et al., 2011, Jaiswal et al., 2013, Robbins et al., 2008). A parallel analysis of mouse MP subsets yielded very similar results (Fig. S6).

### 3.4 Human bona fide CD141^high^XCR1^+^ dermal DCs express MHC-I antigen (cross)-presentation genes

**Fig. S7:**
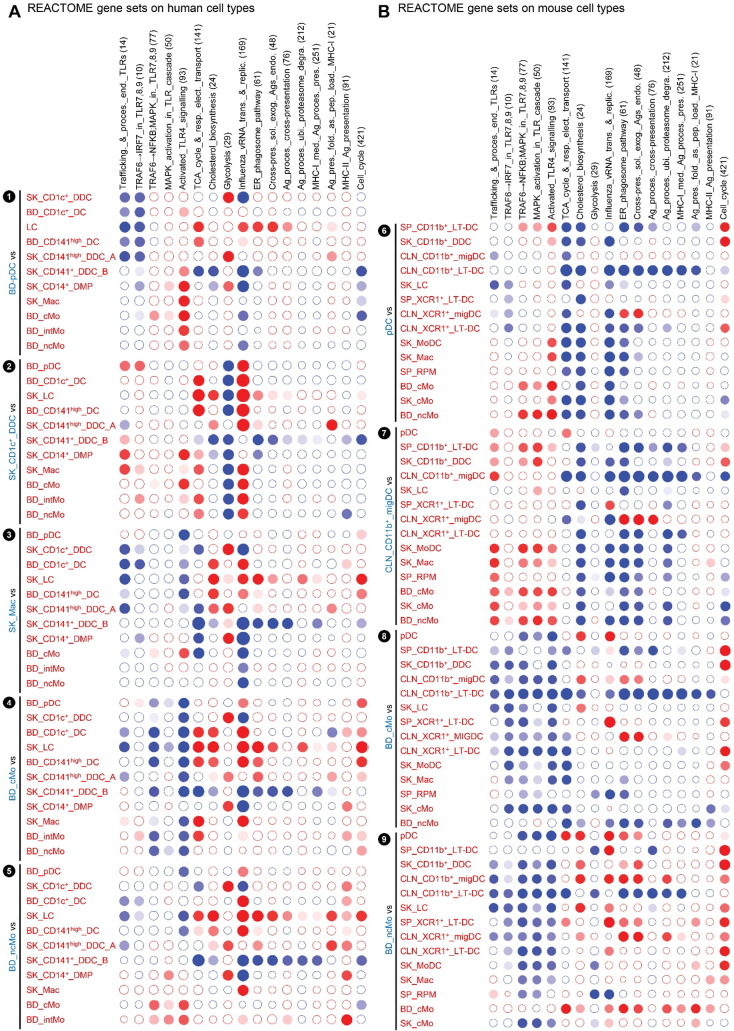
GSEA of selected Reactome GeneSets across human and mouse MP subsets. Additional comparisons between MP subsets to complete the analysis shown in Fig. 7. Selected Reactome GeneSets were assessed for enrichment in all possible pairwise comparisons between MP subsets in the human (A) or mouse (B) compendia. Data are represented as in Fig. 4.

**Fig. 7:**
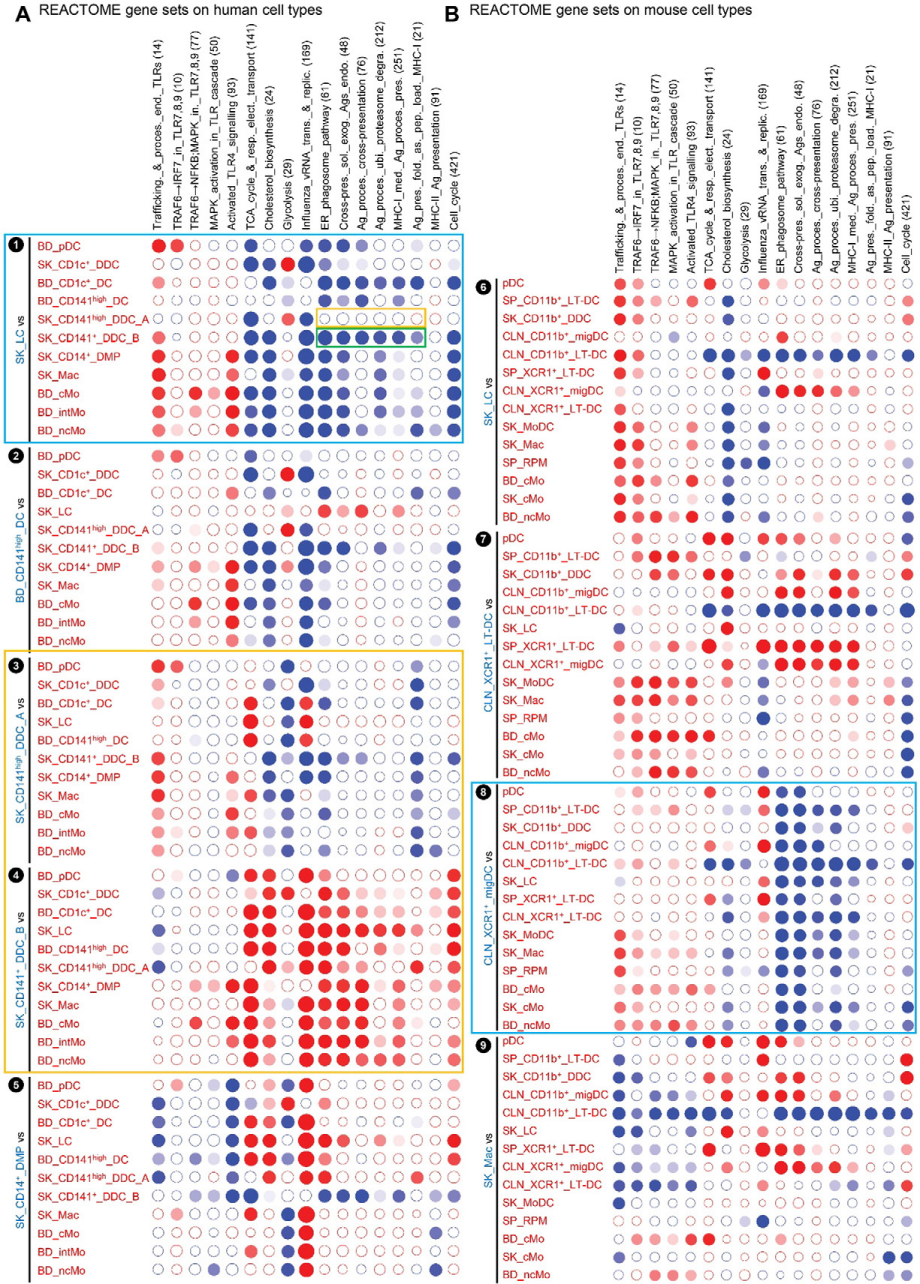
GSEA of selected Reactome GeneSets across human and mouse MP subsets. Selected Reactome GeneSets were assessed for enrichment in all possible pairwise comparisons between MP subsets in the human (A) or mouse (B) compendia. Data are represented as in Fig. 4.

**Fig. 8:**
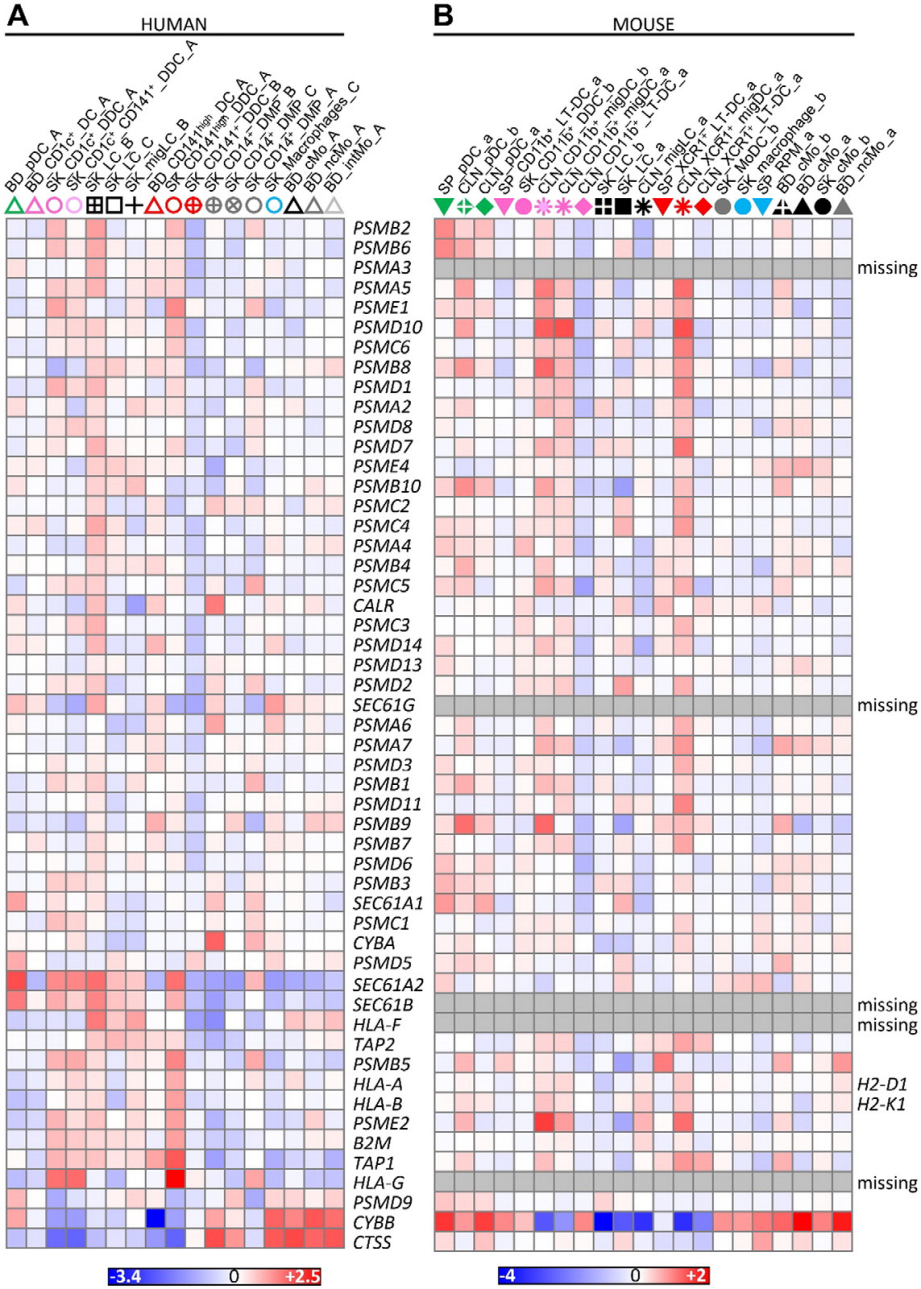
Heatmaps illustrating the expression of MHC-I antigen (cross)-presentation genes. Expression data were collapsed to the median expression across replicates within the human versus mouse compendia. Each cell type is depicted by the same symbol used in the PCA in Fig. 2, with the name of cell types spelled out above.

Upon comparison of the gene expression programs of human SK_LCs with human SK_CD141^+^_DDC_B and CD14^+^_DMPs, it was concluded that human LCs but not human SK_CD141^+^_DDC_B are enriched with MHC-I antigen (cross)-presentation genes (Artyomov et al., 2015). Because we demonstrated above that the SK_CD141^+^_DDC_B from Artyomov et al. and our SK_CD141^high^_DDC_A correspond to different cell types, and because of the discrepancies in previous reports comparing cross-presentation functions of human LCs and dermal CD141^+/high^ DCs (Artyomov et al., 2015, Haniffa et al., 2012), we evaluated the expression of MHC-I antigen (cross)-presentation genes across the whole human MP subset compendium studied here (Figs. 7 and S7). We confirmed that the GeneSets “ER_phagosome_pathway” and “Cross-presentation of soluble exogenous antigens endosomes” were significantly enriched in human SK_LCs when compared not only to SK_CD141^+^_DDC_B (Fig. 7A, ❶, green box) but to all the other human cell populations examined (Fig. 7A, ❶, blue box) but one (Fig. 7A, ❶, orange box). However, importantly, this unique exception corresponded to the SK_CD141^high^_DDC_A. Indeed, both human LCs (Fig. 7A, ❶, blue box) and bona fide human SK_CD141^high^_DDC_A (Fig. 7A, ❸) strongly express “ER_phagosome_pathway” and “Antigen_presentation_folding_assembly_and_peptide_loading_of_class_I_MHC” genes when compared to other human skin MP subsets. This analysis further illustrated the differences between human SK_CD141^+^_DDC_B and SK_CD141^high^_DDC_A, as demonstrated by their differential expression of Reactome GeneSets (subpanels ❸ and ❹ and orange box in Fig. 7A). To interrogate this further, we generated a heatmap of MHC-I antigen (cross)-presentation genes for all human and mouse MP subsets (Fig. 8A). This revealed that most of the MHC-I antigen (cross)-presentation genes were expressed at much higher levels in LCs and cDCs including SK_CD141^high^_DDCs_A than in cells derived from monocytes, which included SK_CD141^+^_DDCs_B. Whereas many of these genes were expressed at similar levels in LCs and SK_CD141^high^_DDCs_A, several were higher in SK_CD141^high^_DDCs_A, including *PSMB5*, *PSMD10*, *PSME2*, *TAP1*, *B2M*, *HLA-A*, *HLA-B* and *HLA-G*.

Hence, among the human MP subsets found in the skin or in the blood, human LCs do stand apart as expressing high levels of the genes associated to MHC-I antigen (cross)-presentation, consistent with similar analyses performed previously (Artyomov et al., 2015). However, high expression of these genes is not a hallmark of human LCs alone and also applies to bona fide human skin CD141^high^XCR1^+^ DDCs. Nevertheless, the expression pattern of the reactome GeneSets associated with MHC-I antigen processing/(cross)-presentation was strikingly similar between human SK_LCs and mouse CLN_XCR1^+^_MigDCs (Fig. 7A, ❶ and Fig. 7B, ❽; blue boxes). Many of the genes associated with MHC-I antigen (cross)-presentation that were selectively expressed at higher levels by human SK_LCs and SK_CD141^high^_DDC_A compared to other human myeloid cell types (Fig. 8A) were also expressed to high levels in mouse CLN_XCR1^+^_migDCs but not by mouse SK_LCs (Fig. 8B), consistent with the differences recently reported between mouse and human LCs (Artyomov et al., 2015).

## 4 Discussion

Recent reports characterized three different cell populations identified as dermal ‘CD141^+^ DCs’ with overlapping phenotypes but each with unique transcriptome profiles, functions, and lineage relationships to other tissue DCs in humans and mice (Artyomov et al., 2015, Chu et al., 2012, Haniffa et al., 2012). This discrepency in the literature has caused confusion in the field regarding how best to identify these cells and define their precise functions. In this study, we aimed to clarify these conflicting reports and to define murine and human skin MP subsets, their intra-species tissue equivalents and inter-species homologs, using comparative genomics. By exploiting public datasets for MP subsets from blood, spleen, skin or cutaneous lymph node of humans and mice, we rigorously identified DC subsets, monocytes and macrophages in these tissues and aligned them across species. We showed here that human dermal CD14^+^ CD141^+^ population (Chu et al., 2012) and dermal CD1a^dim^CD141^+^ cells (Artyomov et al., 2015) are related to monocyte-derived cells and/or macrophages. We also show that the human MP population equivalent to human blood CD141^high^XCR1^+^ DCs are the bona fide CD141^high^XCR1^+^ DDCs (Haniffa et al., 2012) and not LCs as recently claimed (Artyomov et al., 2015). This reaffirms the homologous relationships between human and mouse skin XCR1^+^ DCs and between human and mouse LCs.

In our analysis, both human and mouse LCs transcriptionally resemble cDCs rather than monocytes or monocyte-derived cells. This explains the morphologic and functional similarities between LCs and cDCs supporting the classification of LCs as DCs based on gene expression profiling and function (Artyomov et al., 2015). However, in contrast to cDCs which arise from bone marrow HSCs, LCs develop from yolk sac and fetal liver precursors and are thus developmentally related to tissue-resident macrophages (Hoeffel et al., 2012, Hoeffel et al., 2015). A recent nomenclature attempted to resolve this issue by proposing that MPs should be classified based on a two-stage system: firstly according to ontogeny (level one), and secondly based on their function, location and/or morphology (level two) (Guilliams et al., 2014).

The dichotomy between the developmental and functional attributes of LCs highlights the critical role of the tissue microenvironment in imprinting subset identities on MPs. From birth, the epidermal environment imprints on fetal monocytes a transcriptional program that directs the functional convergence of LCs towards DCs. Similar imprinting also occurs in adult murine skin upon inflammation where distinct LC precursors undergo a differentiation program that culminates in a common end functional phenotype (Chopin et al., 2013, Nagao et al., 2012, Price et al., 2015, Sere et al., 2012). More recently, a reciprocal transcriptomic and functional convergence towards LCs of cells originating from pre-DC was reported, likely resulting from imprinting by the oral mucosa (Capucha et al., 2015). LC subsets arising from different progenitors can be distinguished in the sublingual mucosa of mice, with CD103^+^ CD11b^low^ LCs arising from pre-DCs and CD11b^+^ CD103^−^ LCs from both pre-DCs and monocytic precursors. It could be argued that oral mucosal pre-DC-derived LCs should not be called LCs but rather cDCs due to their ontogeny. However, despite their ontogenic differences, oral mucosal LCs and epidermal LCs share similar immunological functions and transcriptomic signature (Capucha et al., 2015). This supports a significant role of peripheral tissue programming in dictating the final outcome of the functional differentiation of a MP population (Becher et al., 2014, Schlitzer et al., 2015). Peripheral programming ensures that each anatomical niche is populated by MPs with appropriate functions irrespective of their ontogeny. Two recent publications support this notion by demonstrating epigenetic reprogramming within macrophages originating from a given tissue upon adoptive transfer into another tissue (Gosselin et al., 2014, Lavin et al., 2014).

**Fig. S8:**
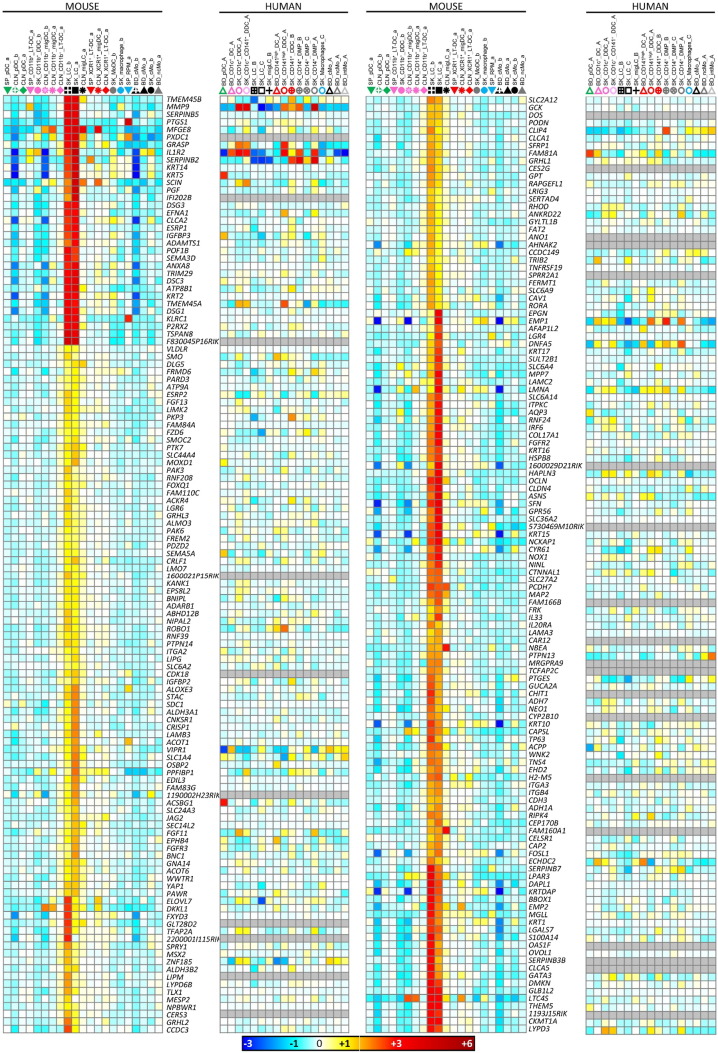
Heatmap showing expression pattern across mouse and human myeloid cell subsets of genes selectively expressed in mouse SK_LCs. Expression data were collapsed to the median expression across replicates within the human versus mouse compendia. Each cell type is depicted by the same symbol used in the PCA in Fig. 2, with the name of cell types spelled out above.

We demonstrate here that human and mouse LCs express a common specific molecular signature and correspond to homologous cell types. Strikingly, however, while mouse LCs are significantly enriched for the human LC gene signature, the reverse is not true (Fig. 4B, ❺, gray box). The mouse LC gene signature is significantly enriched in human SK_CD141^high^_DDC_A and SK_CD1c^+^_DDC when compared to human LCs. However, one should note that the mouse LC gene signature encompasses almost twice as many genes (570) as the human LC gene signature (269). Mouse LCs selectively express many genes in which expression patterns are not conserved between mouse and human skin MPs (Fig. S8). This may explain the distinct biological functions that have been reported between mouse versus human LCs. Mouse LCs were shown to possess tolerogenic (Flacher et al., 2014, Gomez de Aguero et al., 2012, Kautz-Neu et al., 2011, Price et al., 2015, Shklovskaya et al., 2011) rather than immunogenic (Elnekave et al., 2014) functions (reviewed in Igyarto and Kaplan (2013); Malissen et al. (2014)). In contrast, human LCs are generally reported to be immunogenic (Banchereau et al., 2012, Klechevsky et al., 2008, Romano et al., 2012) rather than tolerogenic ((Seneschal et al., 2012, van der Aar et al., 2013); reviewed in Durand and Segura (2015)). Hence, there may be more differences between mouse and human LCs than between other homologous subsets of mouse and human MPs, as was recently proposed (Artyomov et al., 2015). These differences may be accounted for by external environmental factors since, unlike humans, mice are housed in specific pathogen free environment, a condition which may favor the long-term persistence of prenatal LCs. In mice, inflammation can result in partial replenishment of LCs by blood borne precursors which may have unique features compared to prenatally-derived LCs (Chopin et al., 2013, Nagao et al., 2012, Price et al., 2015, Sere et al., 2012). In particular, only prenatally-derived LCs are relatively resistant to irradiation as a consequence of high constitutive expression of the DNA-repair machinery molecule CDKN1A (Price et al., 2015). It is also possible that murine LCs are functionally adapted to fur-bearing mice skin compared to human LCs. Alternatively or in addition, differences in the experimental protocols used for the isolation of MP subsets from mouse versus human skin may also confound comparison of LCs between these 2 species, including differential contamination by other cell types. These differences and heterogeneity may be difficult to evaluate using bulk-population analysis but could be explored with single-cell transcriptome profiling.

Contrary to what was recently reported (Artyomov et al., 2015), we showed that the human skin DCs equivalent to human blood CD141^high^XCR1^+^ DCs are bona fide CD141^high^XCR1^+^ DDCs and not LCs, and we showed that human and mouse LCs are homologous. Why did our interpretation of the analysis of the gene expression profiling data from these authors differ from their own? The discrepancies between our report and Artyomov et al. are primarily methodological in nature. Firstly, the cells isolated as human skin CD141^+^ DDCs in Artyomov et al. were not bona fide CD141^high^XCR1^+^ DDCs but rather cells of the monocytic lineage, as we showed through re-analysis of their transcriptome profile. Secondly, the interpretation by Artyomov et al. of their gene expression profiling data was further confounded by a suboptimal match for cell type composition between the different MP datasets used, namely between the human blood versus skin datasets, and between the mouse versus human skin datasets.

Regarding the nature of the CD141^+^ DDCs from the human “B” dataset, rigorous identification of bona fide human CD141^high^XCR1^+^ dermal DCs is challenging. It requires the use of careful marker combinations for flow cytometry sorting as well as performing a posteriori analyses to ensure of the identity of the cell type isolated (Vu Manh et al., 2015a, Vu Manh et al., 2015b). Unfortunately at present, there isn't a single surface marker that will reliably identify bona fide human CD141^high^XCR1^+^ dermal DCs. CD141 is not unique to these cells but promiscuously expressed by blood and skin CD1c^+^ DCs (Haniffa et al., 2012), a fraction of skin CD14^+^ cells (Haniffa et al., 2012) and MoDCs (Balan et al., 2014). Although XCR1 uniquely identifies these cells at mRNA level in the human, to the best of our knowledge there isn't yet a reliable commercially available antibody against human XCR1. The CD141^+^ DDCs isolated by Artyomov et al. stained with an antibody of unspecified origin but claimed to specifically recognize XCR1 (Artyomov et al., 2015). However, the signal-to-noise ratio of that antibody staining on SK_CD141^+^_DDC_B was very poor, and a similar staining was observed on SK_CD14^+^_DMPs. Hence, the proposed interpretation that XCR1 may not faithfully mark the cross-presenting DC subset in human will need further rigorous experimental testing (Artyomov et al., 2015). Fluorescently-labeled recombinant human XCL1 or XCL2, the ligands for XCR1, specifically stain this receptor on human or monkey XCR1^+^ DCs and do not stain any of the other cell types examined (Balan et al., 2014, Dutertre et al., 2014a), but they are not commercially available. An additional cell surface antigen that could be exploited in identifying human CD141^high^XCR1^+^ dermal DCs is CD11c, because it is expressed at lower levels on these cells as compared to other cDCs, MoDCs and even macrophages (Haniffa et al., 2012). This observation is also recapitulated on in vitro differentiated CD141^high^XCR1^+^ DCs from CD34^+^ cord blood progenitors (Balan et al., 2014). Other antigens such as BTLA, CADM1 or CLEC9A should be used in addition to HLA-DR, CD141, CD11c and negativity for CD14 in characterizing CD141^high^XCR1^+^ DCs (Vu Manh et al., 2015a). The gating strategy we designed and reported when we first identified human CD141^high^ DDCs (Haniffa et al., 2012) was not taken into account when others later attempted to identify, purify and study these same cells (Artyomov et al., 2015, Chu et al., 2012).

**Fig. S9:**
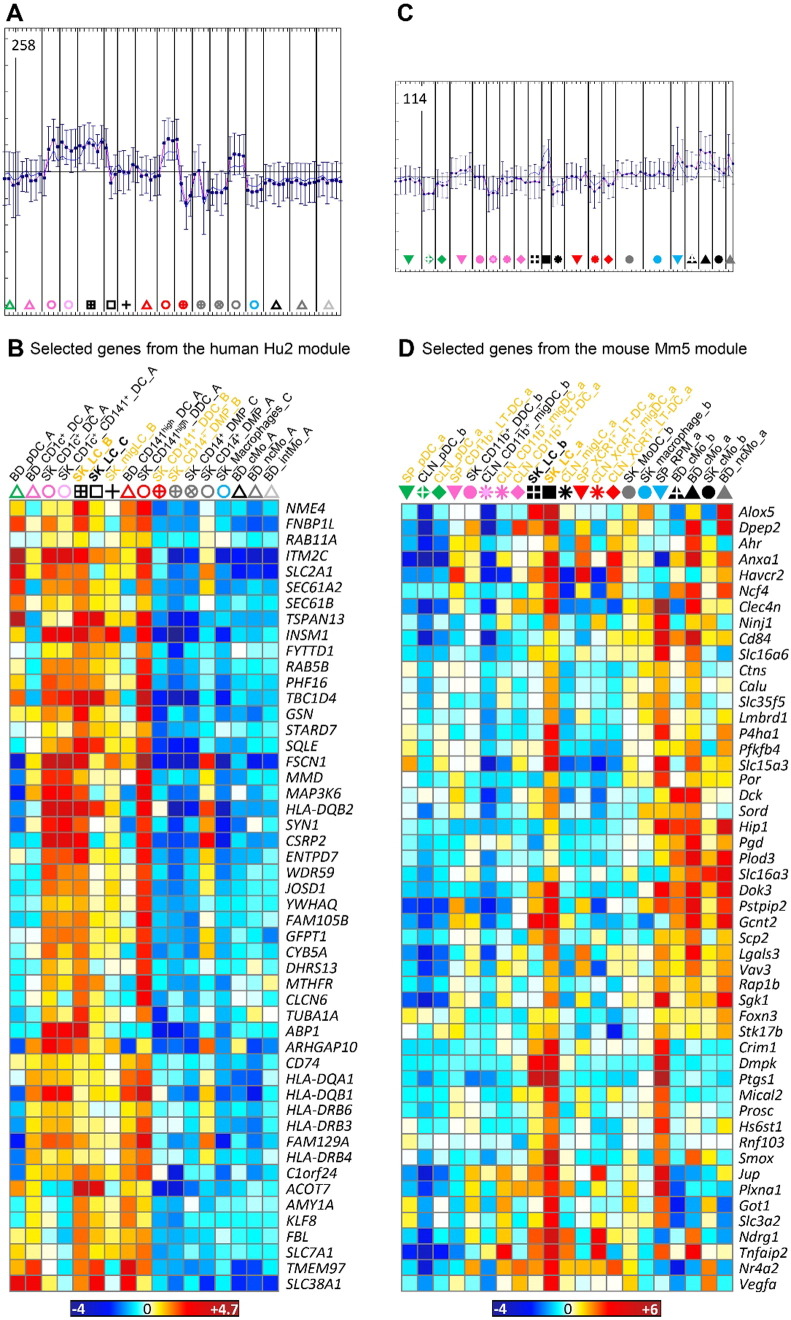
Differences in cell subset composition between the human versus mouse compendia used by Artyomov et al. led to biases in the definition of the gene modules reported to be selectively enriched in human versus mouse LCs. We performed a self-organizing map analysis on the gene modules identified by Artyomov et al. as selectively enriched LCs in human (Hu2 module) or mouse (Mm5 module), in order to cluster these genes based on their expression pattern across the entire human or mouse datasets used here. (A) The second major cluster obtained for the Hu2 module encompassed 258 genes out of the 819 total genes analyzed, and showed higher expression not only in LCs but also in DC subsets as compared to monocytes/macrophages. (B) The individual expression patterns of 50 of these genes are illustrated as a heatmap. (C) The second major cluster obtained for the Mm5 module encompassed 114 genes out of the 265 total genes analyzed, and showed higher expression not only in LCs but also in some monocytes/macrophages as compared to DC subsets. (D) The individual expression patterns of 50 of these genes are illustrated as a heatmap. Above each heatmap, the samples that were initially used by Artyomov et al. to define their gene modules are written in orange while the additional cell types that we used here are written in black. The samples where the genes were expected to be selectively expressed to high levels based on the report from Artyomov et al. are in bold font (SK_LC_B and SK_LC_C for human; SK_LC_a and SK_LC_b for mouse).

In addition to a rigorous isolation strategy, additional a posteriori analyses must be performed to authenticate the identity of the cell population isolated, independently of the antigens used for their phenotypic identification. This can include analysis of their gene expression profiles by microarrays, focused qRT–PCR assays or RNA-Seq, comparing the isolated population with internal positive and negative control populations isolated simultaneously (e.g. other skin MP subsets as well as blood DC subsets), and/or in comparison with existing MP subset transcriptome datasets in publicly available repositories. The microarray transcriptome analysis performed in Artyomov et al. relied on a limited number of MP subsets as comparator populations, with major differences in MP subset composition between the human skin versus mouse datasets and between the human skin versus blood datasets. The mouse dataset used was restricted to mouse CDP-derived DCs and to LCs, without inclusion of monocytes, macrophages or monocyte-derived DCs. In contrast, the human skin dataset used turned out to only encompass monocyte-derived cells and LCs, without inclusion of any CDP-derived DCs. Hence, the gene modules generated through the analyses of these mouse versus human datasets could not be rigorously mapped across species, including for LCs since the reference MP subset populations used to generate the LC-specific signatures are completely different between the two species studied. Specifically, the design of the compendia analyzed by Artyomov et al. led to biasing the human LC gene module for enrichment in genes highly expressed in DC subsets as compared to monocytes/macrophages, and conversely for the mouse LC gene module which as enriched in genes highly expressed in monocytes/macrophages as compared to DC subsets (Fig. S9). A similar problem occurred for the comparison between the human blood versus skin datasets. Surprisingly, the authors of this study (Artyomov et al., 2015) did not directly compare the gene expression profiles of their dermal ‘CD141^+^ DCs’ with the dermal CD141^high^ DC population reported previously (Haniffa et al., 2012). It is unclear why in their analysis Artyomov et al. used only the blood MP from Haniffa et al. but disregarded the skin MP transcriptome data from the same study. In our study, we have tried to match as well as possible the human skin, human blood and mouse compendia. In addition, because each method of gene expression profiling has its own drawbacks and biases, we used different complementary methods to answer our questions, not only module analysis using BubbleGUM but also PCA and hierarchical clustering. This ensured robust interpretation of the results.

We do concur with Artyomov et al., that i) LCs transcriptionally resemble CDP-derived DCs rather than monocyte-derived cells, ii) human but not mouse LCs are enriched in genes linked to MHC-I (cross)-presentation when compared to other monocyte-derived cells, and iii) mouse and human LCs harbor striking differences in their gene expression programs, possibly to a larger extent than other pairs of homologous mouse and human MP subsets. However, our two main findings are contrary to the recent report by Artyomov et al., since we demonstrate that i) human bona fide CD141^high^ DDCs are equivalent to blood CD141^high^ DCs and homologous to mouse XCR1^+^ DCs, and ii) human and mouse LCs are homologous.

Bulk population transcriptomics analysis has enabled enormous progress in the classification and functional interrogation of MPs. However, our study illustrates the critical need for a rigorous design of gene expression compendia in order to accurately map cell populations or biological pathways across tissues or species, which requires using comprehensive datasets as tightly matched as possible across tissue and species for their cell type contents. In addition, our study also highlights the important need for accurate a priori definition of populations during sampling of biological material for RNA extraction, and the need for a posteriori analyses to authenticate the identity of the cell populations isolated such as to determine if the sampling procedure was accurate or needs to be changed (Vu Manh et al., 2015a). In our own studies of MP subsets from pig and sheep, the three-step strategy above allowed us to identify that the cells we had isolated as putative pig homolog of human CD1c^+^ cDCs were not CDP-derived DCs but cells of the monocytic lineage and most likely pig ncMo (Vu Manh et al., 2015b). We anticipate that new unbiased strategies using single-cell transcriptome and proteome analyses will circumvent some of the difficulties with bulk-population studies and provide the resolution to dissect the heterogeneity of MP subsets that underpins the breadth and specificity of immune responses.

The following are the supplementary data related to this article.

Supplementary material. Image 1

## Author contributions statements

M.D. and S.C conceived and developed the original strategy of data analysis with input from T-P.V.M., M.H. and F.G. Bioinformatics analyses were performed and figures generated by S.C., T-P.V.M., R.C. and M.D. The manuscript was written by M.D., F.G. and M.H. with contributions from S.C. and T-P.V.M. B.M. and S.H. provided novel data and critical discussion. All authors reviewed the manuscript.

## Competing financial interest

The authors declare no competing financial interests.
